# Making science of things: Objects and knowledge in, between, and beyond the sciences

**DOI:** 10.1177/00732753261427253

**Published:** 2026-09-01

**Authors:** Tamara Fernando, Brooke Penaloza-Patzak

**Affiliations:** SUNY Stony Brook, USA; The Faculty Center for Transdisciplinary Historical and Cultural Studies, University of Vienna, Austria

**Keywords:** Objects, material culture, environmental history, labor history, history of science, social and economic history, Indigenous history, museums, things

## Abstract

Things and collections of things are among the most ubiquitous forms of scientific evidence, inducing the very concept itself – from the Latin *evidentia*, obvious to the mind or eye – into the physical realm. Things also tend to stick around; that which was acquired for research purposes is eventually transferred to storage, where it either disintegrates, or ages and accumulates. This obduracy of being renders things subject to almost infinite afterlives. Centuries-old evidence can always spark new research questions, perspectives, and methods, therewith exciting the perpetual evolution, branching, and convergence of knowledge and ways of knowing. The process of making things work as scientific evidence is inevitably inexact and messy and requires drawing on ways of working and thinking in and beyond other scientific disciplines. This notwithstanding, conversations about the relationship between objects and scientific knowledge are still largely preoccupied with the epistemic bounds of present-day disciplines and European or Euro-American ways of knowing. The aim of this special issue is to lean into that messiness and think beyond those preoccupations, drawing on new insights from environmental, Indigenous, social, and labor history, among other fields. Contributions foreground how physical objects have been used as evidence to undergird scientific enterprises rather than the disciplines into which those efforts are or have been organized. What emerges are rich histories of how evidence-based ideas and practices have intertwined different fields of knowledge in and far beyond the history of science.

Things and collections of things are among the most ubiquitous forms of scientific evidence, inducing the very concept itself – from the Latin *evidentia*, obvious to the mind or eye – into the physical realm. By virtue of its materiality each thing is a universe of texture, scent, sound, and taste unto itself. Things arrest the senses, can be transported whole or in pieces, and can be sampled from one place to another, and thus arouse the sympathy of those in search of tangible confirmation of that which has been experienced by the ears, nose, eyes, and skin. Things also tend to stick around; that which was acquired for research purposes is eventually transferred to storage, where it either disintegrates, or ages and accumulates. This obduracy of being renders things subject to almost infinite afterlives. Centuries-old evidence can always spark new research questions, perspectives, and methods, therewith exciting the perpetual evolution, branching, and convergence of knowledge and ways of knowing.

The process of making things work as scientific evidence is inevitably inexact and messy and requires drawing on ways of working and thinking in and beyond other scientific disciplines. This notwithstanding, conversations about the relationship between objects and scientific knowledge are still largely preoccupied with the epistemic bounds of present-day disciplines and European or Euro-American ways of knowing.^1^ The aim of this special issue is to lean into that messiness and think beyond those preoccupations, drawing on new insights from environmental, Indigenous, social, and labor history, among other fields. Contributions foreground how physical objects have been used as evidence to undergird scientific enterprises rather than the disciplines into which those efforts are or have been organized. What emerges are rich histories of how evidence-based ideas and practices have intertwined different fields of knowledge in and far beyond the history of science.

Scholarly interest in how physical objects figure into the formation of knowledge commenced in the 1960s among anthropologists and folklorists, and was taken up soon thereafter by historians and sociologists.^2^ Scholars of the history, sociology, and philosophy of science became interested when conversations turned to “object-oriented ontologies” and whether objects too had biographies and “social lives.”^3^ By the 1980s, this once niche area of study was on its way to becoming one of today’s most profoundly interdisciplinary fields of inquiry: material culture studies, which refers to activities as far-ranging as connoisseurship and conservation, museum anthropology and provenance studies.^4^

Today, there are few areas of the humanities and social sciences that remain untouched by material culture studies. The scholarly literature on objects and things spans from philosophy to cultural studies, anthropology, sociology, and history.^5^ It has now been thirty years since Bruno Latour pronounced that social collectives are “woven together out of speaking subjects, perhaps, but subjects to which poor objects, our inferior brothers, are attached at all points,” and nearly twenty since Fiona Candlin and Raiford Guins observed that “everywhere one looks in the academy, objects abound.”^6^ Objects have their own dedicated press series and themed conferences.^7^ Scholars treating objects deploy a range of approaches including phenomenology; autobiography; social, economic, and historical analysis; and philosophical inquiry.^8^ Recent literature in anthropology especially foregrounds how the encounter with the object, in all its tactile, sensual qualities, is, in and of itself, constitutive of thought – hence the proliferation of scholars *thinking through* materiality across fields.^9^

Over the past three decades, historians of science have embraced the “material turn.”^10^ We have investigated the enrolment – acquisition, classification, organization, registration, duplication, exchange, and display – of things in the sciences, insisting that these are value-laden activities that shape the way knowledge is made and disseminated from the local to global scale.^11^ We have adopted the vocabulary and methods of “object lessons,” and interrogated the existence and nonexistence of objects, object agency, the material culture of empirical knowledge, the role of objects in the naturalization of knowledge, and the boundaries between real and epistemic things.^12^ Material culture is becoming a critical point of communion in the interface between the history of science and intellectual history, and scholars working at the intersection of the history of science and museum anthropology are carrying out far-ranging work on the procurement, classification, organization, exchange, and display of things, most recently with a concentration on colonial and postcolonial contexts in particular, establishing that these are value-laden activities that have had a profound impact on the shape of scientific knowledge.^13^

The objects best and longest known to historians of science are arguably those of the metaphysical variety, and as Lorraine Daston has noted, “It is almost impossible for historians of science to speak of the reality of scientific objects without slipping back into epistemological mode.”^14^ This special issue aims to chart an alternate course that moreover attends to how the physical and material reality of things bears upon the formation of science, scientific practice, and knowledge in particular. Contributions build on literature in the history of science by introducing ideas from, among others, social and economic history, Indigenous studies, and environmental history.

Over the past decade, a “neo-materialist” vigor that has likewise taken hold in, among others, environmental history and commodity history, and a situated engagement with matter, now too sits at the center of these studies.^15^ This attention to the vibrant, place-based lives of matter has in turn rekindled a concern for the nonhuman and the animal, and therewith growing awareness about the lessons to be learned from what has historically been considered “other” ways of knowing, Indigenous cosmologies in particular.^16^ At the same time, social histories of artisans and craftspeople coupled with an attention to invisiblized forms of labor have begun asking what and how material-based practices intertwine and transcend knowledge spheres.^17^

These investigations have ushered in a thoroughgoing appreciation that things and collections of things are a fundamental resource in the elision, construction, transformation, and transmission of scientific knowledge over time and across geographic space. In holding fast to disciplinary boundaries, however, the precise ways in which objects traverse the liminal space between established and nascent scientific disciplines as well as diverse knowledge worlds have remained by and large obscured.^18^ Contributions to this special issue explore what happens when we situate things, rather than disciplines, places, or people, at the center of knowledge production.

We know materials matter; now it is time to harness object-centered approaches to build a more inclusive history of science. We must attend to how things are engaged for scientific endeavors not only in, but also between and beyond, science and develop theoretical frameworks capable of comprehending diverse ways of knowing – Indigenous and vernacular in particular – as equally valid and inextricable from the history of science, and capable of elucidating the values embedded in how objects are and have been used as evidence.^19^ To that end, we would also do well to begin unpacking how prerogative-based knowledge has, does, can, and should figure into histories of science.

Patrick Anthony’s article “Nature’s keepers” foregrounds themes of friction, labor, and visibility around the early modern European procurement of scientific objects from nature.^20^ He explores how the Wunder family of Upper Franconia mediated access to and knowledge of nature in the late eighteenth- and early nineteenth-century German lands. By reconstructing a careful history of members of this family, especially its women, Anthony demonstrates that the bones, fossils, and plants that ended up in metropolitan institutions were procured through the logistical and naturalistic knowledge held by village artisans, craftworkers, and other peasants. Citing the now robust literature on the spaces beyond the learned society and university where scientific work takes place (the pub, the kitchen, the home), Anthony begins his history of objects in the village. Things that became scientific objects were initially “strewn among the lumber, turning wheels, planers, and tree-bark” of craftspeople. Drawing inspiration also from Laurel Thatcher Ulrich’s *A Midwife’s Tale*, Anthony turns to social history, asking how historical and anthropological studies of peasant and village life change the narratives of the history of science in early-nineteenth-century Europe.^21^ Though their names were rarely mentioned in scientific publications, craftspeople had a sense of honor about their grasp on learned inquiry: they discovered and named specific caves and were appointed with titles such as “Cave and Naturalia Inspectors.” Anthony blends the scholarship on intermediaries in the history of science with Anna Lowenhaupt’ concept of friction, often taking the latter literally.^22^ That is, he attends to transportation and breakdown along rutty country roads, asking how wagon makers and the upkeep of wheels or carts, and the navigational knowledge needed to traverse country roads and mountain caves, shaped access to specimens. “Viewed from Muggendorf’s market square, self-consciously modern university and museum institutions in Bayreuth and Erlangen rose in reciprocal action with rural projects, otherwise excluded from the story of a liberal bourgeoise public sphere.”^23^ Friction allows Anthony to highlight the artisanal and peasant village “keepers of nature” whose labors and expertise undergirded the procurement of objects that were foundational to the making of the nineteenth-century natural sciences in German lands.

Bruce Buchan and Linda Andersson Burnett’s “Specimens for the ‘science of man’” attends to the literary genre of instructions, and their role in scientific education and in determining what constituted the most legitimate data for the science of man. Instructions played a key role in shaping nineteenth-century notions of race. As they note, “this little-known but profoundly influential pan-European tradition of instructed natural history activated a set of ideas and knowledge practices in a wide range of colonial settings” that “transformed human beings into specimens.”^24^ The authors take as their focus the ethnologist James Cowles Prichard (1786–1848), whose mid-nineteenth-century instructions were published when the genre was over a century old. Burnett and Buchan draw on their book *Race and the Scottish Enlightenment: A Colonial History, 1750-1820* (London: Yale University Press, 2025) to bring the collection of artifacts illustrating “human variety” into conversation with Enlightenment thought more broadly. Skulls played a key role in negotiating the shift in ideas about racial difference away from climatic explanations and toward arguments for the invariable transmission of characteristics of race. Burnett and Buchan’s article sits within narratives of the emergence and consolidation of natural history. In the age of empire and exploration, instructional texts played a key role in, in Joa-Pau Rubiés words, “teaching Europeans how to see the world.”^25^ Instructions stipulated precisely how and what to collect on travels across an increasingly interconnected globe. By placing Prichard within a broader pan-European milieu, as well as that of the “Scottish sciences of man,” Burnett and Buchan consider what it was about the skull as object that distinguished it from, say, hair samples. They explore how changing ideas of race were inflected by and in turn shaped the objects that they relied on for evidence. Pritchard’s dissertation is of special significance here. The essay ends with the posthumous publication of Prichard’s work in John Herschel’s *A Manual of Scientific Enquiry* of 1849, which helped ethnology gain firm footing alongside other scientific disciplines and fundamentally shaped nineteenth-century conceptions about the evidentiary basis for thinking about race.

Raphael Uchôa’s “Amazonian cosmologies, plant-human relations, and the colonial entanglement of Indigenous artifacts” addresses Indigenous knowledge and objects in the history of science with recourse to theoretical insights from Indigenous philosophy. Drawing on emerging conversations on decolonial curatorship led by Indigenous thinkers and in museum studies, Uchôa’s aim is to “go beyond questions of access, inclusion or provenance.”^26^ He analyzes the politics of display surrounding Amerindian masks and botanical specimens from the Amazon basin at the Science Museum at the University of Coimbra and Kew Gardens, asking what happens to the “political, historical, or cosmological dimensions” that shaped the “original meaning” of Indigenous objects. For Uchôa, “text-based” methods alone are inadequate for such work. His intervention is rooted in the admission of oral traditions as a source of information for writing object-based histories of science that do not reduce Indigenous knowledge to mere “symbolic footnotes or cultural folklore.” The objects at the University of Coimbra were originally assembled by Portuguese naturalist Alexandre Rodrigues Ferreira during his *Viagem Filosófica* (1783–92). Uchôa builds on insights founded on the words of Yanomami shaman Davi Kopenawa, who describes museums as sites of “cosmological captivity,” as well as Eduardo Viveiros de Castro’s notion of “counter-anthropology.” Uchôa begins by articulating the Indigenous skills that “formed the unacknowledged epistemic infrastructure for European natural history,” botanical knowledge, for instance, and canoe-building, and river navigation.^27^ As he notes, Coimbra’s museum and its organization of objects was reformed in the eighteenth century to espouse “an empirical curriculum aligned with Enlightenment ideals.” Using Kopenawa’s writings, Uchôa shows how a Jurupixuna mask, for instance, is not simple an “object or representation” but instead an “animated form that condenses relations between humans, animals, and spirits.”^28^ In so doing, he helps bring the conversation begun by Gosden, Larson, and Petch nearly two decades ago full-circle, demonstrating that objects in museum collections are not passive artifacts, nor merely of value for the history of science, but moreover and just as significantly links and agents in the formation of Indigenous knowledge and cosmologies.^29^ In the second half of the paper, he addresses fragments of *Baninsteriopsis caapi*, traditionally used to prepare the psychoactive brew ayahuasca, which were collected from the Rio Negro by English botanist Richard Spruce. Uchôa reads these fragments alongside Tukano people’s cosmologies to offer a different understanding of the bounds that separate the vegetal and human worlds. Attending to objects through Indigenous cosmologies offers us, in this account, a view of the museum as “site of ongoing epistemic struggle.” In order for material-centered studies in the history of science to advance, we must acknowledge and grapple with linguistic and conceptual gaps, as well as the ethos of access.

Brooke Penaloza-Patzak’s “Sense and the science of nineteenth-century environmental determinism” turns to the use of objects as data to draw the concept of intersubjectivity into dialogue with the history of the senses and the environment.^30^ This article considers U.S. naturalist William Healey Dall (1845–1927) in nineteenth-century Beringia and his observations based on collections made there of human and other-than-human life. As Chief of Explorations in Russian America for the Western Union Telegraph Expedition’s Scientific Corps, Dall’s investigations commingled natural history, ethnography, and archaeology. Disciplinary boundaries do not constrain Penaloza-Patzak’s inquiry; instead, she follows objects across them. She discusses how and why Dall postulated that the environs of Beringia affected the development of its invertebrates, situates such work in relation to the more general nineteenth-century coming into being of the concept of environment, and walks readers through how Dall then employed methods and theories from the natural sciences to theorize about the origins and development of Indigenous American culture. She shows how Dall’s physical and sensory experience of place shaped what was posited as “objective knowledge” about how the environment affected organic beings. By taking Dall’s subjective experience of place seriously, Penaloza-Patzak is able to deploy his diaries and sensory experience “as a tool for situating that subjective knowledge alongside the practical and theoretical aspects of [his] practice.”^31^ In this treatment of both human and cultural development, she responds to Sadiah Qureshi’s call for a more thoroughgoing “‘peopling’ of natural history,” following objects and place-based knowledges across disciplines.

Finally, Kuang-Chi Hung and Leo Chu’s article “A Tale of Two Genera” asks what knowledge can be gleaned from missing or incomplete specimens. They begin with one of the foremost “founders” of American botany, Asa Gray (1810–88), whose discovery of the plant *Shortia galicifolia* is so renowned that it has been accorded its own U.S. postal stamp. Beginning with a specimen Gray encountered at the Jardin des plantes in Paris, they unravel a transnational network of interlocutors and scholars responsible for Gray’s ultimate “discovery” of the plant. It is incomplete and fragmentary specimens, as well as their accompanying notes and marginalia, that prompt Hung and Chu to ask “whether Grey’s quest is merely an American botanical story.”^32^ Instead, they trace knowledge about *Shortia galicifolia* from St. Petersburg to Japan, China, and Paris, modeling how specimens lead us through knowledge worlds, such as that of nineteenth-century Japan and botanist Itō Keisuke 伊藤圭介 (1803–1901), who identified and depicted the plant in correspondence sent to Russian botanist Carl Johnann Maximowicz (1827–91). When Gray first came across the specimen in Paris, he thought it had come from mountains in North Carolina; “It shall be christened *Shortia*, to which we will stand as godfathers,” he wrote to American botanist John Torrey (1796–1873) Ibid., p. 257. Hung and Chu draw on scholars such as Federico Marcon, Naohide Isono and Makoto Tanaka, and Masuzō Ueno, working on the history of science in Japan to highlight and build on the international engagements of nineteenth-century Japanese *honzō* scholars. By tracing these connections through object-based marginalia and specimens, they are able to shed light on a much more global history of botanical knowledge, with deep roots in other epistemic traditions in China and Japan, which intersected with American botanical knowledge in the nineteenth century.

As these synopses suggest, our contributors engage a range of objects from human to other-than-human: rare plants, cave bear bones, human skulls, a zoomorphic mask, mollusks, and shell middens. The disciplines they speak to include ethnology, botany, malacology, anthropology, natural history, and the earth sciences, and more than one discipline frequently appears in the same article. In terms of chronology, the earliest period treated is the 1760s, while Uchôa’s contribution takes us up to the present day. In geographical scope, the articles are wide-ranging. Moreover, by focusing on the movement of objects over great distances, the contributions destabilize clear models of the center–periphery: schematic drawings of plants may move from Japan to Boston, and mollusk shells from the Bering Strait to Washington. These objects were derived, procured, and stolen from, among others, mountain caves, tropical rainforests, and arctic straits. Things move between herbaria, museums, universities, and field sites, and each contribution emphasizes different facets of the procurement, curation, analysis, use, and display of objects. In treating different phases in the existence of objects of science, each essay offers a distinct yet complementary vantage point. That is, some authors address the role of instructions as a template for how objects are expected to function as part of a collection; others the physical, infrastructural, and material environment from which collection was made; while others spend more time with the display of things. Each contribution to this special issue thus helps shed light on different facets in the history of the effort to “make science of things.”

The extraction and movement of things is central to several of the essays. Buchanan and Andersson Burnett’s article brings the genre of instruction manuals into dialogue with the objects they were intended to acquire. Collection instructions established what kinds of objects should be collected and how, and as such formed an early juncture in the conceptualization of objects as evidence. In attending to instruction as a template, Buchanan and Andersson Burnett provide a strong starting point for thinking about how objects were supposed to work as evidence in the scientific imagination. Their contribution underscores how race-making in this period relied on skulls as evidence, dovetailing with other articles that treat scientific hierarchies of human difference, including Penaloza-Patzak’s and Uchôa’s.

Environments and cultures of origin oftentimes mattered as much as the materiality of objects themselves. How could one preserve or document the soft bodies of a glassy nautilus of the Bering Strait, or the delicate leaves of *Shortia galacifolia*? Indeed, the original physical, material, and social environment of origin from which things sprang had the potential to shape both their epistemic status and scientific discourses about them. In fact, as several contributors show, these environments continued to matter even after objects were disembedded from their original contexts. Penaloza-Patzak’s article considers how discourses and personal experience of Beringian environs shaped how Dall viewed biotic – and increasingly cultural – development in the Bering Strait. This entanglement of both the human and natural sciences through objects emerges in other articles, including Uchôa’s.

Following objects also allows room for the emergence of global histories of scientific practices and networks. Chu and Hung’s article uses marginalia and preserved specimens of *Shortia galacifloia* housed today in Boston to illustrate a transregional network of botanical knowledge moving between the United States, Japan, and Russia. Our contributors’ essays reference predictable figures in the history of science across botany, natural history, and the earth sciences – Carl Linneaeus, Alexander von Humboldt, Samuel George Morton, and Louis Agassiz – and also make space alongside them for less widely known figures. Anna Graf (b. 1777), for example, who acted as a cave inspector, natural history guide, and owner of a natural history cabinet in Muggendorf; or Aleutian Island native Norman Walker (dates unknown), who assumed the role of sometime guide, interpreter, and collections procurer for the Russian American Telegraph Expedition of 1865–7. This also includes contemporary individuals, such as Yanomami scholar, shaman, and activist Davi Kopenawa, who insists that, among other things, the act of looking is never neutral.

The obduracy of things means that they persist across time, continuing to both generate new meanings and to distort older ones, leading in some cases to the dissociation of knowledge. For instance, in confronting contemporary collections and archives, many of our authors contemplate what is lost in the transfer to a museum and how objects in collections are often “suspended from the histories and cosmologies that once gave them meaning.” Uchôa asks us to consider the museum in terms of “the weight of its collections – and the silences they carry.” Buchanan and Andersson Burnett explain that while skulls became “incontrovertible data from the dead,” they were also “rootless artifacts of knowledge” with little indication of the histories of colonial dispossession and violence that accompanied their procurement. Museology, Anthony shows, recorded the name of the famed naturalist rather than the innkeeper who aided him, and thus wrote out working-class contributions to object gathering: “learned collections were dissociated from rural keepers.”^33^ Just as objects facilitate the continual generation of knowledge, they also result in the warping and disassociation of other knowledges.

In using and deploying objects as evidence, scientific practitioners, and later museum curators, were making epistemic choices. Our special issue makes clear that things always inhabit multiple knowledge worlds, and that the portals between them never permanently close. Instead, the position of an object between epistemic orders is negotiated over and over again. Collectively, attending to how things and collections of things have been involved in the making, transfer, loss, and transformation of specialized knowledge and ways of knowing draws our attention to the spaces in, between, and beyond the natural and human sciences from the eighteenth century to today. In exploring how and why objects are selected and repurposed for scientific investigation, transformed into specimens and then data, and involved in the transmission of ways of knowing and ordering, historians can reconstruct the evolution of knowledge, cultivation of expertise, and underpinnings of authority. Throughout this issue, contributions focus on logics of possession, the assembly of object collections as an extractive and essentializing enterprise, and on the use of ideals like “reason,” “science,” and “truth” for race-making and asserting hierarchies of human and other-than-human difference. Working and thinking with objects in focus, in other words, offers us new ways of appraising the thing we call science.

## Footnotes

1. For special issues and collected volumes that take a more capacious view, bringing different fields into conversation, see Marianne Klemun, Marina Loskutova, and Anastasia Fedotova, “Skulls and Blossoms: Collecting and the Meaning of Scientific Objects as Resources from the 18th to the 20th Century,” *Centaurus* (special issue) 60 (2018): 231–7; Felix Driver, Mark Nesbitt, and Caroline Cornish (eds.), *Mobile Museums. Collections in Circulation* (London: University College London Press, 2021); and Ina Heumann, Anne MacKinney, and Rainer Buschmann, “Introduction: The Issue of Duplicates,” *The British Journal for the History of Science* (special issue) 55, no. 3 (2022): 257–78.

2. For foundational works, see especially: Henry Glassie, *Pattern in the Material Folk Culture of the Eastern United States* (Philadelphia, PA: University of Pennsylvania, 1968); Jules David Prown, “Mind in Matter: An Introduction to Material Culture Theory and Method,” *Winterthur Portfolio* 17, no. 1 (1982): 1–19; and James Clifford, *The Predicament of Culture: Twentieth-Century Ethnography, Literature, and Art* (Cambridge: Harvard University Press, 1988).

3. Michel Callon, “Some Elements of a Sociology of Translation: Domestication of Scallops and the Fishermen of St Brieuc Bay,” *The Sociological Review* 32, no. 1 (1984): 196–233; Arjun Appadurai, *The Social Life of Things: Commodities in Cultural Perspective* (Cambridge: Cambridge University Press, 1986); Bruno Latour, *Science in Action: How to Follow Scientists and Engineers Through Society* (Cambridge: Harvard University Press, 1987).

4. The *Museum Anthropology* journal was established in 1976. Today, there are myriad graduate programs dedicated to material culture studies and auxiliary topics; among the earliest established were the Winterthur Museum (1952) and Bard Graduate Center (1993).

5. For overviews of this literature see Dan Hicks and Mary C. Beaudry (eds.), *The Oxford Handbook of Material Culture Studies* (Oxford: Oxford University Press, 2010); and Fiona Candlin and Raiford Guins, “Introducing Objects,” in Fiona Candlin and Raiford Guins (eds.), *The Object Readers* (Oxford: Routledge, 2009), pp. 1–24.

6. Bruno Latour, *Aramis or the Love of Technology*, trans. Catherine Porter (Cambridge, MA: Harvard University Press, 1996), p.vii; Candlin and Guins, “Introducing Objects,” p.3 (note 5).

7. Objekt series, published by Reaktion Books 2004-present, <https://press.uchicago.edu/ucp/books/series/RB-O.html> (accessed 12 December 2026).

8. Candlin and Guins, “Introducing Objects,” p.2 (note 5).

9. Tim Ingold, *Making: Anthropology, Archaeology, Art and Architecture* (Abingdon, Oxon and New York, NY: Routledge, 2013).

10. On the material turn, see: Anita Guerrini, “The Material Turn in the History of Life Science,” *Literature Compass* 13 (2016): 469–80; Hans-Jörg Rheinberger, “The ‘Material Turn’ and the ‘Anthropocenic Turn’ from a History of Science Perspective,” in Gabriele Dürbeck and Philip Hüpkes (eds.), *The Anthropocenic Turn. The Interplay Between Disciplinary and Interdisciplinary Responses to a New Age* (London: Routledge, 2020), pp.27–36.

11. E.g., Timothy Lenoir and Cheryl Lynn Ross, “The Naturalized History Museum,” in Peter Galison and David Stump (eds.), *The Disunity of Science: Boundaries Context and Power* (Stanford, CA: Stanford University Press, 1996), pp.370–97; Anke te Heesen, *The World in a Box. The Story of an Eighteenth-Century Picture Encyclopedia* (Chicago, IL: Chicago University Press, 2002); Anke te Heesen, “News, Paper, Scissors,” in Lorraine Daston (ed.), *Things That Talk* (New York, NY: Zone Books, 2004), pp.297–327; Anke te Heesen, *Der Zeitungsausschnitt. Ein Papierobjekt der Moderne* (Frankfurt/Main: Fischer Taschenbuch Verlag, 2006); Chris Gosden, Fran Larson, and Alison Petch, *Knowing Things: Exploring the Collections at the Pitt Rivers Museum 1884-1945* (Oxford: Oxford University Press, 2007); Sarah Byrne et al. (eds.), *Unpacking the Collection: Networks of Material and Social Agency in the Museum* (New York, NY: Springer-Verlag, 2011); Brooke Penaloza-Patzak, “An Emissary from Berlin: Franz Boas and the Smithsonian Institution, 1887–88,” *Museum Anthropology* 41, no. 1 (2018): 30–45; James Poskett, *Materials of the Mind. Phrenology, Race, and the Global History of Science, 1815-1920* (Chicago, IL: Chicago University Press, 2019); Brooke Penaloza-Patzak, “Of Scientists and Specimens: Early Anthropology Networks in and Between Nations and the Natural and Human Sciences,” in David McCallum (ed.), *The Palgrave Handbook on the History of the Human Sciences* (Singapore: Palgrave Macmillan, 2022), pp.1651–78; Driver et al. (eds.), *Mobile Museums* (note 1); Heumann et al., “The Issue of Duplicates” (note 1); Anne Greenwood MacKinney, *Nature on Paper. Documenting Science in Prussia, 1770-1850* (Pittsburgh, PA: Pittsburgh University Press, 2024). These works must moreover be understood as part of a conversation with scholars in other fields, especially museum anthropology, in particular Chris Gosden and Yvonne Marshall, “The Cultural Biography of Objects,” *World Archaeology* 31, no. 2 (1999): 169–78; Chris Gosden, Fran Larson, and Alison Petch, *Knowing Things: Exploring the Collections at the Pit Rivers Museum 1884-1945* (Oxford: Oxford University Press, 2007); Beatrix Hoffmann, *Das Museumsobjekt als Tausch- und Handelsgegenstand. Zum Bedeutungswandel musealer Objekte im Kontext der Veräußerungen aus dem Sammlungsbestand des Museums für Völkerkunde Berlin* (Berlin: Berliner Münster Lit, 2009); Gwyneria Isaac, “Whose Idea Was This? Museums, Replicas, and the Reproduction of Knowledge,” *Current Anthropology* 52, no. 2 (2011): 211–33; Candace Greene, “Material Connections: The ‘Smithsonian Effect’ in Anthropological Cataloguing,” *Museum Anthropology* 39, no. 2 (2016): 147–62; Hannah Turner, *Cataloguing Culture: Legacies of Colonialism in Museum Documentation* (Vancouver, BC: UBC Press, 2020); Catherine A. Nichols, *Exchanging Objects. Nineteenth-Century Museum Anthropology at the Smithsonian Institution* (New York, NY: Berghahn Books, 2021).

12. Bruno Latour and Steve Woolgar, *Laboratory Life. The Social Construction of Scientific Facts* (Beverly Hills, CA: SAGE, 1979); Lorraine Daston (ed.), *Biographies of Scientific Objects* (Chicago, IL: University of Chicago Press, 2000), which includes, among others, Bruno Latour’s “On the Partial Existence of Existing and Nonexisting Objects,” in Loraine Daston ed. *Biographies of Scientific Objects* (Chicago: University of Chicago Press, 2000), pp. 247–69; Lorraine Daston (ed.), *Things that Talk: Object Lessons from Art and Science* (New York, NY: Zone Books, 2004); Bruno Latour, *Reassembling the Social: An Introduction to Actor Network Theory* (Oxford: Oxford University Press, 2005); Pamela H. Smith, Amy R. W. Meyers, and Harold J. Cook, *The Material Culture of Empirical Knowledge* (Ann Arbor, MI: University of Michigan Press, 2014); Hans-Jörg Rheinberger, *Towards a History of Epistemic Things: Synthesizing Proteins in the Test Tube* (Stanford, CA: Stanford University Press, 1997); Hans-Jörg Rheinberger, “A Reply to David Bloor: ‘Toward a Sociology of Epistemic Things’,” *Perspectives on Science* 13, no. 3 (2005): 406. One of the most controversial and yet enduring frameworks for thinking-with-objects in the history of science has of course been actor–network theory, or ANT. ANT’s afterlives in the history of science have ranged from histories of cinchona bark in the British Empire to notions of water in global history as an ecological “object.” See Rohan Deb Roy, *Malarial Subjects: Empire, Medicine, and Nonhumans in British India, 1820-1909* (Cambridge: Cambridge University Press, 2017); on calls to “ecologize” history that draw from Latour, see Tamara Fernando, Felice Physioc, and Alexis Rider, “Flows of History,” *Past & Present* 260, no. 1 (2023): 1–31.

13. On the history of science and intellectual history, see, e.g., Rheinberger, “The ‘Material Turn’” (note 10); John Tresch, “Cosmologies Materialized: History of Science and History of Ideas,” in Darrin M. McMahon and Samuel Moyn (eds.), *Rethinking Modern European Intellectual History* (Oxford Academic, online ed., 2014), pp.153–172. On collections in the history of science, see: te Heesen, “News, Paper, Scissors” (note 11); te Heesen, *The World in a Box* (note 11); te Heesen, *Der Zeitungsausschnitt* (note 11); Penaloza-Patzak, “An Emissary from Berlin” (note 11); Driver et al. (eds.), *Mobile Museums* (note 1); Penaloza-Patzak, “Of Scientists and Specimens” (note 11); and MacKinney, *Nature on Paper* (note 11).

14. See especially Latour and Woolgar, *Laboratory Life* (note 12); Bruno Latour, “Where Are the Missing Masses? The Sociology of a Few Mundane Artifacts,” in Wiebe E. Biijker and John Law (eds.), *Shaping Technology/Building Society: Studies in Sociotechnical Change* (Cambridge, MA: MIT Press, 1992), pp.225–58; Rheinberger, *Towards A History of Epistemic Things* (note 12); and, for the quote, see Lorraine Daston, “The Coming into Being of Scientific Objects,” in Lorraine Daston (ed.), *Biographies of Scientific Objects* (Chicago, IL: University of Chicago Press, 2000), p.14; see also Martin Heiddegger, *Frage nach dem Ding* (Tübingen: Max Neimeyer Verlag, 1968).

15. Jane Bennet, *Vibrant Matter: A Political Ecology of Things* (Durham, NC: Duke University Press, 2010); Timothy LeCain, *The Matter of History: How Things Create the Past* (Cambridge: Cambridge University Press, 2017); Christian Jacob, “*Lieux de savoir*: Places and Spaces in the History of Knowledge,” *KNOW: A Journal on the Formation of Knowledge* 1, no. 1 (2017): 85–102.

16. See Bathsheba Demuth’s discussion of “vibrant matter” against indigenous cosmologies in the Introduction to *Floating Coast: An Environmental History of the Bering Strait* (New York, NY: W.W. Norton & Company, 2019); Robin Wall Kimmerer, *Braiding Sweetgrass: Indigenous Wisdom, Scientific Knowledge, and the Teachings of Plants* (Minneapolis, MN: Milkweed Editions, 2013); Helen Tilley, “Global Histories, Vernacular Science, and African Genealogies; or, Is the History of Science Ready for the World?” *Isis* 101, no. 1 (2010): 110–19; Projit Bihari Mukharji, “Vishalyakarani as Eupatorium Ayapana: Retro-Botanizing, Embedded Traditions, and Multiple Historicities of Plants in Colonial Bengal, 1890–1940,” *The Journal of Asian Studies* 73, no. 1 (2014): 65–87; Tresch, “Cosmologies Materialized” (note 13); Helen Anne Curry, “Taxonomy, Race Science, and Mexican Maize,” *Isis* 112, no. 1 (2021): 1–21; Shira Shmuely, “Curare: The Poisoned Arrow That Entered the Laboratory and Sparked a Moral Debate,” *Social History of Medicine* 33, no. 3 (2020): 881–97; Anja Kanngieser and Zoe Todd, “From Environmental Case Study to Environmental Kin Study,” *History and Theory* 59 (2020): 385–93; Efram Sera-Shriar, “From Museamization to Decolonialization: Fostering Critical Dialogues in the History of Science with a Haida Eagle Mask,” *British Journal for the History of Science* 56, no. 3 (2023): 309–28; Raphael Uchôa, “‘Savage Knowledge’ Ethnosciences, and the Colonial Ways of Producing Reservoirs of Indigenous Epistemologies,” *Journal of Social Ontology* 10, no. 2 (2024); Raphael Uchôa, Staffan Muller-Wille, and Harriet Mercer, “Science and Its Others: Histories of Ethnoscience,” *History of Anthropology Review* 48 (2024).

17. On invisibilized labor, see Jenny Bangham, Xan Checko, and Judith Kaplan (eds.), *Invisible Labour in Modern Science* (London, Rowman and Littlefield, 2022); and on the other mentioned themes, see Pamela Smith’s *The Body of the Artisan* (Chicago: University of Chicago Press, 2004); Pamela O. Long, *Artisan/Practitioners and the Rise of the New Sciences, 1400-1600* (Corvallis, OR: Oregon State University, 2011); Heidi Hausse, “The Locksmith, the Surgeon, and the Mechanical Hand. Communicating Technical Knowledge in Early Modern Europe,” *Technology and Culture* 60, no. 1 (2019): 34–64; Lucia Dacome et al., “Unruly Objects – Material Entanglements in the Arts and Sciences,” *Nuncius* 35 (2020): 461–70.

18. For an early outlier in this respect, see Timothy Lenoir, *Instituting Science. The Cultural Production of Scientific Disciplines* (Stanford, CA: Stanford University Press, 1997); and for more recent work that transcends disciplinarily defined histories, see especially Bernard Lightman and Efram Sera-Shriar, *Victorian Interdisciplinarity and the Sciences. Rethinking the Specialization Thesis* (Pittsburgh, PA: Pittsburgh University Press, 2024), and Fabian Kraemer, Kärin Nickelsen, and Dana von Suffrin, “Botany and the Science of History: Nature, Culture, and the Origins of Civilization, ca. 1850-1900,” *Isis* 113, no. 1 (2022): 45–62.

19. Crucial impulse in this respect comes from Lisa Onaga’s “Reclaiming Turtles All the Way Down (TAWD): Plural Practices of Animal Cosmologies” working group in the Artifacts, Action, Knowledge Department at the Max Planck Institute for the History of Science in Berlin. See e.g. Lisa Onaga, Stamatina Mastorakou, and Lakshmi Pradeep Rajeswary, “Manners of Reckoning: Epistemology, Cosmology, Ontology, and Worlding,” research project, Max Planck Institute for the History of Science, 2024-, (accessed 12 December 2025).

20. Patrick Anthony, “Nature’s keepers and the economy of earthly objects,” *History of Science* 64 (2026): 191–225.

21. Laurel Thatcher Ulrich, *A Midwife’s Tale: The Life of Martha Ballard, Based on Her Diary, 1785–1812* (New York: Vintage Books, 1991).

22. Anna Lowenhaupt Tsing, *Friction: An Ethnography of Global Connection* (Princeton, NJ: Princeton University Press, 2005).

23. Anthony, “Nature’s keepers,” p. 196 (note 20).

24. Bruce Buchan and Linda Andersson Burnett, “ Specimens for the “science of man”: Skulls, race, and instructions in James Prichard’s Enlightenment ethnology,” *History of Science* 64 (2026): 226–246, p. 227.

25. Joa-Pau Rubiés, “Instructions for Travellers: Teaching the Eye to See,” *History and Anthropology* 9, no. 2–3 (1996): 139–190, p. 140. Cited in Buchan and Andersson Burnett, “Specimens,” p. 230 (note 24).

26. Raphael Uchôa, “Amazonian cosmologies, plant-human relations, and the colonial entanglement of Indigenous artifacts,” History of Science 64 (2026): 297-325., p. 302.

27. Uchôa, “Amazonian cosmologies,” p. 304 (note 26).

28. Ibid., p. 308.

29. Gosden et al., *Knowing Things* (note 11).

30. Brooke Penaloza-Patzak, “Sense and the science of nineteenth-century environmental determinism,” *History of Science* 64 (2026): 272–296.

31. Ibid., p. 287.

32. Kuang-Chi Hung and Leo Chu, “ A tale of two genera; or, how plant specimens from Japan contributed to the greatest legend in American botany,” *History of Science* 64 (2026): 247–271, p. 251.

33. Uchôa, “Amzonian cosmologies,” p. 306 (note 26); Buchan and Andersson Burnett, “Specimens,” p. 228 (note 24); Anthony, “Nature’s keepers,” p. 217 (note 20).

